# Evaluating the Effects of Frequency of Subthalamic Nucleus Deep Brain Stimulation on Postural Control in Parkinson’s Disease: A Case-Series Study

**DOI:** 10.3390/jcm13216357

**Published:** 2024-10-24

**Authors:** Nazlı Durmaz Çelik, Aslı Yaman Kula, Elif Göksu Yiğit Tekkanat, Müge Kuzu Kumcu, Mehmet Yanardağ, Serhat Özkan

**Affiliations:** 1 Department of Neurology, Faculty of Medicine, Eskişehir Osmangazi University, 26040 Eskişehir, Turkey; dr.egyigittekkanat@gmail.com (E.G.Y.T.); scozkan@gmail.com (S.Ö.); 2 Department of Neurology, Faculty of Medicine, Bezmialem Foundation University, 34093 İstanbul, Turkey; dr.asliyaman@gmail.com; 3 Department of Neurology, Faculty of Medicine, Lokman Hekim University, 06530 Ankara, Turkey; muggykuzu@gmail.com; 4 Research Institute for Individuals with Disability, Anadolu University, 26470 Eskişehir, Turkey; yanardagm@gmail.com

**Keywords:** postural control, deep brain stimulation, subthalamic nucleus, Parkinson’s disease, sensory organization test

## Abstract

**Background/Objectives**: Subthalamic nucleus deep brain stimulation (STN-DBS) is a standard treatment for motor complications in Parkinson’s disease (PD). Its impact on axial symptoms is still not fully understood. This study aimed to quantitatively evaluate the effect of frequency changes within the therapeutic window on postural control performances of individuals with PD who underwent bilateral STN-DBS. **Methods**: Postural control was assessed using Computerized Dynamic Posturography with randomized DBS frequency parameters, low (60 Hz), high (130 Hz), and very high (180 Hz), across six sensory organization test (SOT) conditions. **Results**: Twenty PD participants with a mean age of 61.2 ± 10.1 years were included. There were no differences in equilibrium scores of SOT conditions between 60, 130, and 180 Hz frequencies (*p* > 0.05), except the SOT6 score (*p* = 0.003), where 60 Hz showed better equilibrium performance in SOT6, indicating an advantage in postural control when visual cues are disturbed. **Discussion:** Low-frequency settings (60 Hz) in STN-DBS may benefit those who rely heavily on visual cues while ineffectively using somatosensory and vestibular inputs. **Conclusions:** A tailored approach to the DBS frequency setting could optimize postural stability and reduce fall risk in these patients. Future research is needed to explore these mechanisms to enhance therapeutic strategies.

## 1. Introduction

Parkinson’s disease (PD) is a progressive and chronic neurodegenerative disorder characterized by an array of motor symptoms, such as resting tremor, bradykinesia, rigidity, and postural instability, as well as non-motor symptoms, including sleep problems, autonomic dysfunction, cognitive impairment, or mood disorders. The degeneration of dopaminergic neurons in the substantia nigra region implicated in motor control predominantly gives rise to these symptoms [1]. Of these, postural instability and gait disturbance are some of the most disabling motor symptoms of PD, particularly in the late stages of the disease. In the eventual progressive phase, treatment-refractory motor signs and symptoms include postural instability and falling in 87% (with fractures in 35%) [2]. These axial symptoms increase the risk of falls, impair mobility, and reduce quality of life, making their control a key priority in PD [3]. The efficacy and safety of subthalamic nucleus deep brain stimulation treatment for PD (STN-DBS) are well established when considering motor symptom management, such as tremors, dyskinesia, and wearing-off phenomena refractory to pharmacotherapy [4]. The electrodes are implanted in the subthalamic nucleus for stimulation to suppress abnormal neural activity and reduce insuppressible motor symptoms from the STN-DBS circuit. Although the anti-tremor and motor complication effects of STN-DBS are well recognized, its efficacy on axial symptoms, particularly postural instability, and gait difficulties, remains to be proven and is still being researched. A few studies suggest that the improvement in postural control and gait are time-limited, such that these benefits may eventually wane or deteriorate over the long term [5,6,7], especially in a medication state [8]; however, a few recent studies report long-term beneficial effects on axial symptoms [9,10]. Due to these contradictory results, no consensus has been reached on STN-DBS outcomes about postural instability and gait disturbances among patients with STN-DBS, which signifies that examining DBS parameters further for improvement in axial symptoms is worthwhile. Recent studies have shown that in DBS programming, frequency may be a critical parameter and that it is as effective as the stimulation voltage/current and pulse width in DBS outcomes [11,12]. For instance, a meta-analysis showed that <100 Hz and >100 Hz stimulations of the STN have distinct effects on different PD axial motor symptoms when the patient is in a medication state, including freezing of gait (FOG), one of the most disabling hardly completely alleviated motor symptoms from treatments even when all targets are integrated [13]. Although high-frequency stimulation (often ~130 Hz) has been a standard approach to treat PD motor symptoms, recent studies suggested that low-frequency stimulation offers more significant benefits for axial symptoms. In contrast, high-frequency stimulation may be superior in reducing tremors and rigidity [11,14]. This is with the varied effects of DBS frequency on motor and axial symptoms that we know are possible. Abnormal postural control has been shown in PD as a fundamental component of mobility and balance, the impairment of which results in considerable disability and impaired quality of life [5]. Thus, it is suggested that different frequencies of STN-DBS should attenuate postural control differently in this manner, with a lower frequency likely providing more effective residuals to stabilize posture. Therefore, this study hypothesizes that different frequencies of STN-DBS will have various effects on postural control in individuals with PD. This study aims to quantitatively evaluate the impact of frequency changes on postural control performances of individuals with PD who underwent bilateral STN-DBS.

## 2. Materials and Methods

This case-series study was conducted at Eskisehir Osmangazi University Faculty of Medicine. Patients with PD and STN-DBS were recruited from the movement disorder outpatient clinic between May 2021 and June 2022. They were assessed under different DBS frequency conditions in the Research Institute for Individuals with Disability, Department of Physical Therapy and Rehabilitation Clinic of Anadolu University, Eskisehir. The study was approved by the Ethical Committee (Approval no./date: 2021034-2021/5) of Eskisehir Osmangazi University Medical Faculty. This study was carried out by Code of Ethics of the World Medical Association (Declaration of Helsinki) 1975, as revised in 2013. All patient details were de-identified to prevent the possibility of patient identification. The reporting of this study conforms to STROBE guidelines [15].

### 2.1. Participants

Before participating in the research, every participant gave written consent after being fully informed according to ethical guidelines, and all were given a full explanation of the study methodology, risks, and potential benefits. We included patients who had a diagnosis of Parkinson’s disease (PD) that was confirmed using the UK Brain Bank Criteria [16]. All participants had previously undergone bilateral subthalamic nucleus deep brain stimulation (STN-DBS) and were required to have a Mini-Mental State Examination (MMSE) score higher than 25 [17]. This cut-off was selected to allow all participants with adequate cognitive function to understand and follow the instructions for the assessment of postural control.

Individuals with other neurological (such as stroke, multiple sclerosis, or other neurodegenerative diseases), musculoskeletal (e.g., severe arthritis or orthopedic conditions detrimental to gait or postural control), and cognitive or psychological conditions causing gait and postural control disruption were excluded. Patients whose postural disturbances were unresponsive to levodopa were also excluded.

**Figure 1:**
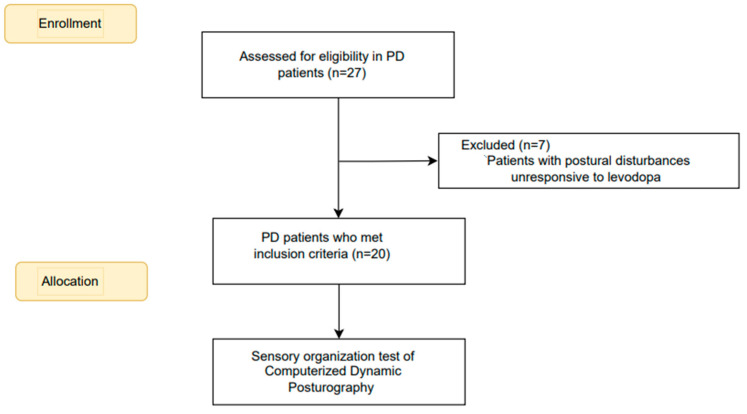
Flowchart for recruitment and allocation of PD patients.

Before the study, a power analysis was performed to establish an appropriate sample size with sufficient statistical power. The power analysis indicated that a sample size of 20 participants was adequate to detect meaningful differences in postural control measures. Figure 1 elaborates on a detailed flowchart regarding recruitment. Additionally, the screening and placement of eligible participants identify how they were recruited, screened, and enrolled in the study.

### 2.2. Procedures

Demographic (age, height, weight) and clinical characteristics (disease duration, time after surgery, modified Hoehn and Yahr stage, levodopa equivalent daily doses) [17] of the participants were collected. Cognitive functions were evaluated with MMSE [17]. Participants were assessed with the Turkish version of the Unified Parkinson’s Disease Rating Scale Part III (UPDRS III) [18] and the Freezing of Gait Questionnaire (FOGQ) [19,20].

Postural control was evaluated by a sensory organization test (SOT) of Computerized Dynamic Posturography (CDP) (Neurocom^®^ Balance Manager System Software V9.2, Natus MEdical Inc., Seattle, WA, USA.) [21]. Six conditions (three static and three dynamic) assessed the participant’s ability to maintain an upright stance without excessive swaying, integrating somatosensory, visual, and vestibular inputs. The three static conditions with a stable force plate were as follows: SOT1, participants stood with their eyes open; SOT2, participants stood with their eyes closed; and SOT3, participants received incongruent visual feedback through a moving visual environment that did not match their physical sway, simulating sensory conflict. The three dynamic conditions with a rotated force plate about the ankle joint axis in proportion to the participant’s spontaneous anterior–posterior (AP) sway were as follows: SOT4, participants stood with their eyes open; SOT5, participants stood with their eyes closed; and SOT6, participants received incongruent visual feedback with a sway-referenced visual surround. In the sway-referenced visual surround conditions, both the visual surround and the platform moved in sync with the participant’s body sway, creating a challenging postural environment, and the participant’s visual environment rotated around the ankle joint axis in proportion to their spontaneous sway, like the movement of the platform in dynamic conditions. The equilibrium score indicates the participant’s sway within expected angular stability limits during each SOT condition. Participants with little sway achieved scores near 100, while those approaching their stability limits scored near 0. During the SOT, participants were evaluated three times in each condition to obtain an equilibrium score ranging between 0 and 100, of which 100 indicated the best postural balance, and the final equilibrium score for each condition was calculated by taking the average of three scores.

Furthermore, a composite equilibrium score was also calculated by taking the average of all scores of six conditions. In addition to the equilibrium score, CDP measured “initial alignment” and “strategy” scores during each SOT condition. The “Initial alignment” score shows a participant’s initial center of gravity (COG) position before each SOT trial. The force plate also measured vertical forces that, along with the participant’s height, were used to calculate the COG angle. In CDP, the change in the COG angle about the ankle joint for both feet was measured in real time [21]. If the participant’s COG is behind or to the left of their center of foot support, the initial alignment values are reported as unfavorable; if the participant’s COG is in front or to the right of their center of foot support, the initial alignment values are positive. The participant’s “Strategy” score reflects their use of ankle and hip movements to maintain equilibrium during each 20 s trial. A score near 100 indicates that the participant primarily uses the ankle strategy to maintain equilibrium, while a score near 0 shows that the participant primarily uses the hip strategy (NeuroCom^®^,Neurocom Balance Manager System Software V9.2, Natus MEdical Inc., Seattle, WA, USA, 2011). The composite balance score was calculated by averaging the scores obtained from the six SOT conditions.

The participants in this study were evaluated by a group of specialists, including a neurologist and physical therapist experienced in movement disorders. The assessments were performed in the ‘on’ state when patients had the best motor function because of optimal antiparkinsonian drug treatment and deep brain stimulation (DBS) settings. It is reached about an hour after a dose of levodopa, the primary drug used to treat PD motor symptoms. This timing assured the maximal response potential of each participant, reducing between-subject variability resulting from motor state fluctuations [22].

The test stimulation condition was like the screening condition, except that the frequency (60 Hz, 130 Hz, or 180 Hz) was randomized first among participants to avoid any bias inherent to the sequence of frequency changes in DBS and, thus, DBS effects. In addition, randomization dilutes the possibility of giving rise to factors such as fatigue or adaptation that could bias postural control assessments. We enforced a 10 min wait time in between each frequency adjustment to make sure that the effects of the previous frequency setting would subside. This was precautionary in nature to prevent carryover effects from the preceding stimulation setting affecting responses at each frequency tested [23]. We chose this short period because previous studies have demonstrated that frequency effects occur rapidly after exposure [24].

**Figure 2:**
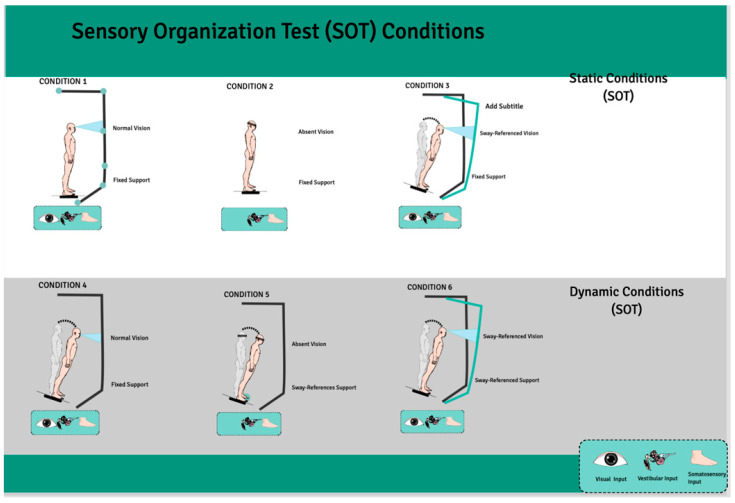
Three static and three dynamic sensory organization test (SOT) conditions of Computerized Dynamic Posturography [26].

The postural control of the participants was assessed by the sensory organization test (SOT) of Computerized Dynamic Posturography (CDP). There were three static conditions and three dynamic conditions that challenged the different sensory modalities (vision, somatosensory, and vestibular systems) involved in keeping balance. The static conditions consisted of participants standing on a fixed platform (SOT1, 2, 3), and the dynamic conditions consisted of movements of the platform being standardized to occur in time with the participant’s sway (SOT4, 5, 6), challenging their postural stability. Figure 2 includes a summary of the six conditions/rules used to manipulate sensory inputs to challenge static and dynamic postural control [25]. This method provides a sensitive evaluation of the changes in postural stability during standing within different sensory environments, with very small DBS frequency variation having detectable effects on both static and dynamic balance performance. Three static and three dynamic sensory organization test (SOT) conditions of Computerized Dynamic Posturography are summarized in Figure 2.

### 2.3. Statistical Analyses

Mean ± standard deviation and Median values were given in descriptive statistics for continuous data, and number and percentage values were given in discrete data. The Kolmogorov–Smirnov test was used to examine the conformity of the data to normal distribution. Friedman’s 2-way ANOVA analysis was used to compare the Equilibrium values at 60 Hz, 130 Hz, and 180 Hz in the SOT positions of the patients, and Friedman’s Multiple Comparisons (post hoc) test was used to determine the time between the measurements. The IBM SPSS for Windows 20.0 (SPSS Inc., Chicago, IL, USA) program was used in the evaluations, and *p* < 0.05 was accepted as the statistical significance limit. The data from the research were added as Figure 2.

## 3. Results

### 3.1. Demographics and Clinical Characteristics of Participants

The study included the data of 20 participants with PD (9 women and 11 men) with a mean age of 61.2 ± 10.1 years. The mean duration of disease was 13.7 ± 5.0 years, and the mean duration of STN-DBS surgery was 3.7 ± 2.2 years with a range of 1–9 years. Participants had a Hoehn and Yahr stage range between 1 and 3 and UPDRS III scores between 22 and 29. Demographics and clinical characteristics are given in Table 1.

### 3.2. Static Postural Control

No difference was found between the Equilibrium values at 60 Hz, 130 Hz, and 180 Hz in the SOT1, SOT2, and SOT3 positions (*p* > 0.05) (Table 2, Figure 1).

### 3.3. Dynamic Postural Control Results

**Figure 3:**
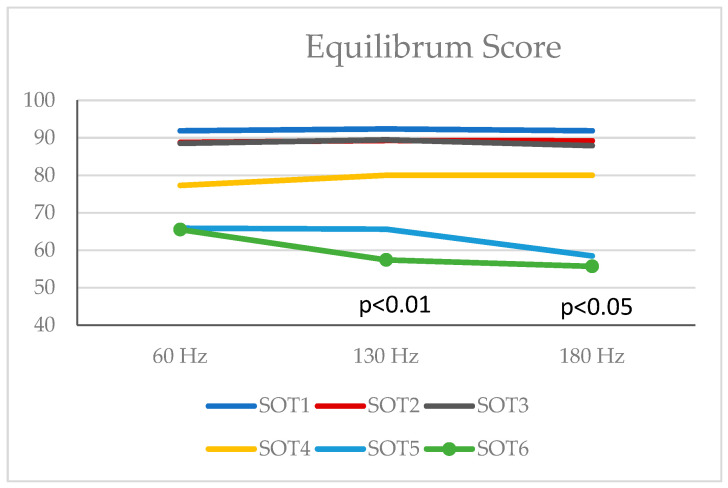
Equilibrium values at 60 Hz, 130 Hz, and 180 Hz in SOT1, SOT2, SOT3, SOT4, SOT5, and SOT6 positions.

There were no differences in equilibrium scores of SOT4 and SOT5 conditions. There was a difference between the Equilibrium values at 60 Hz, 130 Hz, and 180 Hz in the SOT6 position (*p* < 0.01). According to Friedman’s Multiple Comparisons (post hoc) test results, there was a difference between Equilibrium values at 60 Hz and 130 Hz and between Equilibrium values at 60 Hz and 180 Hz. Equilibrium values at 130 Hz were lower than the Equilibrium values measured at 60 Hz (*p* = 0.004), and Equilibrium values at 180 Hz were lower than the Equilibrium values measured at 60 Hz (*p* = 0.028). There was no difference between Equilibrium values at 130 Hz and 180 Hz (*p* > 0.05) (Table 2, Figure 3).

According to the post hoc analysis, participants with 60 Hz showed significantly better equilibrium performance in SOT6, which is disturbed by visual, somatosensory, and undisturbed vestibular systems. The participants had similar COG and strategy scores between 60, 130, and 180 Hz frequencies (*p* > 0.05 for all scores). There was no difference between the Composite Scores at 60 Hz, 130 Hz, and 180 Hz (*p* > 0.05).

## 4. Discussion

This study may be one of the most structured studies to evaluate the effect of several frequency parameters on postural sway in patients with PD with STN-DBS. We assessed the effect between low-frequency stimulation at 60 Hz, high-frequency stimulation at 130 Hz, and very-high-frequency stimulation at 180 Hz on postural control in patients with PD who underwent bilateral STN-DBS, using objective measurements. Results from the current study revealed that the performance at low frequencies was similar to that at higher frequencies. Values of postural sway did not differ in selected frequency parameters. The only exception was SOT6—when the patients’ eyes were open, with a sway-referenced platform and swayed visual surroundings with disadvantaged somatosensory and visual systems in which individuals mostly rely on visual cues—performing better at low frequencies (60 Hz) when compared with higher frequencies (130, 180 Hz). This suggests that the mechanisms of STN-DBS frequency modulation may involve more complex interactions with sensory integration processes, potentially affecting neuroplasticity in sensory pathways. Future research could explore these mechanisms further to enhance therapeutic strategies. The DBS frequency settings for improvement in axial symptoms of PD are still controversial. An increasing number of reports have explored the use of low-frequency stimulation on gait symptoms [6,12,27,28] and postural control [6].

Three DBS frequencies, 60, 130, and 180 Hz, were chosen in our study based on clinical experience and well-established data showing that low–high and very-high-frequency stimulation have distinct effects on motor and axial symptoms of Parkinson’s disease (PD) [24]. Axial symptoms such as postural instability and freezing of gait, which often challenge conventional high-frequency settings, have improved significantly at low-frequency stimulation (60 Hz). For example, in a study for freezing of gait and postural control, much-improved outcomes were found with 60 Hz compared to 130 Hz stimulation, especially in patients with medication-refractory axial symptoms [14]. Moreover, these findings were confirmed by a new study, which demonstrated additionally in low-frequency DBS that balance was improved, and gait disturbances were reduced [29].

However, 130 Hz continues to be used as the benchmark for treating motor symptoms of tremor, rigidity, and bradykinesia in many studies [6]. We included 180 Hz based on recent evidence that very high frequencies might modulate separate neural pathways, making it a candidate for ameliorating specific symptoms. In utilizing these three frequencies, our study planned to provide a broad assessment of the impact on postural control spanning both conventional and somewhat nascent DBS settings. The intent of this strategy was to capture the widest range of possible therapeutic effects and target both motor and axial (postural) symptoms in PD subjects.

### 4.1. Static Postural Control

This study found no effect of different stimulation frequencies (60 Hz, 130 Hz, and 180 Hz) on static postural control in patients with STN-DBS for PD. The 60 Hz stimulation condition is at least consistent with the holy war literature on low frequency (below 100 Hz) stimulation, which has found conflicting evidence for the ability of this type of sound to improve stabilization. For example, in a prior study, Xie et al. (2015) reported improvement in axial symptoms (swallowing, balance, and postural instability) by low-frequency stimulation. Their results demonstrated significant improvement in these axial symptoms by 60 Hz STN-DBS compared with 130 Hz stimulation according to UPDRS III axial subscores [14]. Nevertheless, these findings referred mainly to axial symptoms, and they were not attributed exclusively to the static component of postural control.

On the other hand, Vallabhajosula et al. considered a broader scope of stimulation (60 Hz or higher than 100 Hz), and the resulting effect on posture and gait. The study found no statistically different results in static postural control or gait characteristics between 9 and 50 Hz [6]. This indicates that static postural control is mainly based on visual and somatosensory input, besides dynamic motor control. This could be one of the reasons why no significant effect was seen for frequency changes in our study or in the other study. The present findings suggest that the use of stable visual and somatosensory cues for static postural control in patients with PD may not be sensitive to frequency modulation across low, high, or very high ranges of stimulation through STN-DBS.

### 4.2. Dynamic Postural Control

In Parkinson’s disease, dynamic postural control faces a more complex process that includes somatosensory and visual inputs together, as well as vestibular control and motor coordination.

The results for dynamic stability reported by studies investigating different STN-DBS stimulation settings are almost entirely subjective, with clinical scales or accelerometer-based harmonic ratio measurements, each of which may lack objectivity and variability across studies [6,12,14,30,31]. Dynamic postural control can be evaluated objectively with accelerometer-based harmonic ratio measurement. Higher harmonic ratios were considered to represent greater gait rhythmicity and dynamic postural stability. Dynamic postural control was evaluated by calculating the harmonic ratio with an accelerometer. Nevertheless, there are still discrepancies in results, and these may be related to the use of various methods to evaluate dynamic postural control between studies.

A study conducted this way reported that lower frequency was better with dynamic postural control. However, in the current study, the method gives information about dynamic postural control with a more objective, quantitative, and reliable method. Another study revealed that the dynamic postural control at low frequency was similar to the higher-frequency stimulations. In that study, only eyes-opened and -closed evaluations were performed [6]. Similarly, the current study showed no difference between the frequencies of SOT4 (eyes open) and SOT5 (eyes closed) performances. In addition, the present study showed that 60 Hz patients had significantly better equilibrium performance in SOT6, which is a condition with a disturbed visual and somatosensory system and undisturbed vestibular system. The dynamic postural controls of the participants are only impaired when they are challenged. Previous studies have not evaluated the effect of very-high-frequency stimulation on dynamic postural control. Conversely, in Vallabhajosula et al.’s study, dynamic postural stability was not significantly different when low-frequency and high-frequency stimulation were compared. Dynamic postural control was measured less comprehensively in their study (eyes-open and eyes-closed conditions on a stable surface), and their findings indicated no frequency-specific effects [6]. The inconsistency in these findings may be due to the measurement methods—subjective clinical scales or objective metrics like accelerometry.

Dynamic postural control was evaluated with increased precision and reliability in the current study across multiple frequencies (60 Hz, 130 Hz, 180 Hz) of assessments of postural stability. Differences in specific conditions emerged, unlike those found in static postural control. Overall, subjects exhibited a significantly greater equilibrium score in SOT6 when presenting 60 Hz over any other frequency, under conditions where the visual and somatosensory systems were degraded, and vestibular input was predominant for postural stability. This suggests that low-frequency stimulation might provide some specific benefits in dynamic postural control, particularly when sensory feedback is disrupted, or the vestibular system is a primary balance cue.

In addition, to the best of our knowledge, this is one of the first studies (if not only) to have looked at very-high-frequency stimulation (180 Hz) on dynamic postural control. However, previous studies have mostly neglected the effect of high frequencies (>130 Hz) on postural control in PD, and the resultant gap obstructs a complete understanding of DBS frequency effects. The results of the present study showed no substantial disparity in dynamic postural control outcome measures between very high frequencies and those that are considered as high frequencies, and frequencies higher than 130 Hz may not add to a betterment in dynamic stability. Nevertheless, great caution should be taken in interpreting these results due to various limitations of the study, such as the relatively short duration of frequency application per test condition. Short- and long-term outcomes for high- and very-high-frequency stimulations of postural control in Parkinson’s disease need to be explored.

In the current study, the effect of not only low-frequency but also very-high- and high-frequency stimulation was examined. Dynamic postural control in very-high-frequency stimulation is unchanged compared to high-frequency stimulation. In brief, we think that the reason for the controversial results of previous studies in the measurement methods is that when evaluated objectively, the difference between frequencies regarding postural control is negligible. While our findings suggest minimal postural disturbance across frequencies, clinicians should consider the study’s limitations, including the short duration of frequency application. Longer stimulation periods may yield different outcomes.

### 4.3. Limitations

The current study has several limitations. The small sample size (*n* < 30) potentially affects the generalizability of the results to a larger population. A 10 min adaptation period was used among the three frequency conditions so a more extended period could evoke various results in the dependent variables. All measurements were performed in an ON-medication state, so an interaction of various frequency stimulations with medication could stimulate some effects on the postural control. Furthermore, by applying more than one measurement, it could be better to interpret the results and the change in adults’ postural control levels in different test conditions and frequencies. The results in the current study do not reflect the long-term effects of the various frequency conditions; they include acute impact on postural control.

Another limitation of this study is that we did not change the stimulation amplitude/voltage, which would have allowed us to compare frequency and amplitude more directly. Several previous studies [18] have shown that both voltage and frequency changes can each independently influence outcomes such as freezing of gait and postural control. However, future studies should consider voltage/amplitude as a variable to elucidate the interaction between these changes.

There is a further limitation in that patient selection may vary, as the response to low-frequency stimulation could greatly depend on individual disease trajectories and comorbidities. Although we applied strict inclusion criteria, future studies should include more patients in different stages to evaluate the effectiveness of low-frequency STN-DBS on a variety of patients’ profiles. Furthermore, our study did not evaluate the effects of low-frequency stimulation on non-motor symptoms such as verbal fluency, which reportedly improves with low-frequency STN-DBS in a few studies.

In addition, while the current study contributes significant information about postural control under different frequencies of STN-DBS, the lack of a non-stimulated control group with which to compare an outcome to a baseline in stability indeed determines that future investigations should use such controls to understand fully the consequences of DBS on postural stability.

## 5. Conclusions

Results from the current study indicated that though low-frequency stimulation (60 Hz) produced similar results compared to high- (130 Hz) and very-high-frequency (180 Hz) stimulation for postural control, the low frequency may be helpful for individuals with PD who are over-reliant on visual cues and ineffective use of somatosensory and vestibular cues. In addition to the sensory organization test, various motor control tests should be applied to verify low-frequency stimulation’s short- and long-term effects on the target group. Importantly, this finding underscores the possibility that low-frequency stimulation may have some clinical utility in patients who have become visually dependent yet show a diminished ability to flexibly use somatosensory and vestibular inputs for balance control and modulation. This reliance on somatosensory function may compromise their overall posture control and may render these patients suitable targets for low-frequency stimulation as an intervention to improve balance.

Although the sensory organization test was used to measure the influence of various types of brightness stimuli on postural control, we recommend that future studies include other motor control tests. These should include tests necessary to look at both the immediate and long-term effects of low-frequency stimulation on this group. A full battery of such tests can help establish whether the added benefit conferred by low-frequency stimulation is reproducible and abundant enough over time to warrant its consideration as an alternate modality for treating axial postural control in persons with PD.

## Figures and Tables

**Table 1:** Demographics and clinical characteristics of participants.

Patients’ Characteristics	
	*n* = 20
Age (years), Mean ± SD	61.2 ± 10.1
Female, *n* (%)	9 (45)
Duration of disease (years), Mean ± SD (Min–Max)	13.7± 5.0 (8–18)
BMI (kg/m^2^), Mean ± SD	27.4 ± 2.9
Time after surgery (years), Mean ± SD (Min–Max)	3.7 ± 2.2 (1–9)
UPDRS III score, Mean ± SD	25.8 ± 2.2
MMSE score, Mean ± SD	27.7 ± 1.7
LEDD (mg/day), Mean ± SD	1165.2 ± 646.9
FOGQ, Mean ± SD (Min–Max)	8.0 ± 0.85 (2–15)
Age (years), Mean ± SD	61.2 ± 10.1(38–75)

BMI: Body mass index; LEDD: Levodopa equivalent daily dose; MMSE: Mini-Mental State Examination; UPDRS III: Unified Parkinson’s Disease Rating Scale Part III; FOGQ: Freezing of Gait Questionnaire; SD: Standard deviation; Min–Max: Minimum–maximum.

**Table 2:** Sensory organization Equilibrium test scores in three treatment conditions.

Frequency		60 Hz	130 Hz	180 Hz	*p*-Value
		Mean ± SD Median (Min–Max)	Mean ± SD Median (Min–Max)	Mean ± SD Median (Min–Max)	
**Equilibrium**	SOT1	91.9 ± 2.4 92 (84.3–95.3)	92.4 ± 2.5 92.9 (85.6–95.6)	91.9 ± 2.2 92 (86.0–95.3)	0.695 ^b^
	SOT2	88.8 ± 3.8 88.8 (77.0–96.0)	89.3 ± 3.3 89.3 (82.3–94.6)	89.2 ± 2.6 89.4 (83.6–94.0)	0.781 ^b^
	SOT3	88.5 ± 5.0 89.0 (73.3–94.0)	89.5 ± 3.7 90.0 (82.3–95.6)	87.9 ± 4.4 88.4 (76.6–93.6)	0.161 ^b^
	SOT4	77.3 ± 12.1 81.5 (48.3–90.0)	80.0 ± 8.9 81.1 (60.3–90.3)	80.0 ± 8.5 80.0 (58.3–92.0)	0.580 ^b^
	SOT5	65.9 ±8.9 65.8 (50.6–86.0)	65.6 ± 13.5 66.6 (26.6–89.0)	58.5 ± 17.3 64.1 (17.0–80.3)	0.358 ^b^
	SOT6	65.5 ± 14.5 64.0 (33.3–90.3)	57.4 ± 21.6 61.6 (14.0–92.0)	55.7 ± 18.6 55.3 (16.7–86.6)	**0.003 **^b^ *
**Composite Score**		74.5 ± 12.0 77.0 (39.0–90.0)	74.9 ± 10.9 76.0 (46–92)	72.9 ± 9.7 75.0 (55.0–88.0)	0.307 ^b^

Mean ± SD and Median (min–max) values were used as descriptive statistics. ^b^: Friedman’s 2-way ANOVA. *: *p* < 0.01. SOT: Sensory organization test; SD: Standard deviation; min–max: Minimum–maximum.

## Data Availability

The original contributions presented in the study are included in the article; further inquiries can be directed to the corresponding authors. We can confirm that the data supporting the findings of this study are available and can be shared (Figure 2).
